# Abnormal origin of the right posterior segmental bronchus: case report and literature review

**DOI:** 10.1186/s13019-023-02296-0

**Published:** 2023-07-12

**Authors:** Tao Xu, Hui Ye, Wenshu Chen, Zhongwen Jin, Changjin He

**Affiliations:** 1 Department of Thoracic and Cardiac Surgery, Ningde Municipal Hospital Affiliated to Ningde Normal University, Ningde Fujian, 352100 China; 2 Department of Thoracic Surgery, Fujian Provincial Hospital, Shengli Clinical Medical College of Fujian Medical University, Fuzhou Fujian, 350001 China

**Keywords:** Three-dimensional reconstruction, Segmentectomy, Variations, Right upper lobe bronchus, Lung cancer

## Abstract

**Background:**

With the increasing availability of chest computed tomography (CT), the detection of small pulmonary nodules has become more common, facilitating the development of lung segmental resection. However, anatomical variations of the bronchi are common, particularly in the right upper lobe of the lung.

**Case presentation:**

We report a case of thoracoscopic resection of the posterior segment of the right upper lobe of the lung. Preoperatively, the nodule was believed to be located in the superior segment of the right lower lobe. However, intraoperative exploration revealed that the nodule was located in the posterior segment of the right upper lobe, further showing that the bronchi of the posterior segment of the right lung opened into the bronchus intermedius. The procedure was completed uneventfully. Postoperative retrospective three-dimensional (3D) reconstruction of the lung CT images confirmed that the bronchi of the posterior segment of the right upper lobe originated from the bronchus intermedius.

**Conclusions:**

This rare case highlights the importance of 3D reconstruction to guide accurate segmentectomy in patients with anatomic variations.

## Background

The increasing application of video-assisted thoracoscopic surgery for lung surgery requires accurately determining lung anatomy to prevent severe complications. Postoperative lung function is better preserved during anatomic segmentectomy than lobectomy, with the former garnering great interest [1]. However, because of the anatomical complexity of the lung, segmentectomy is more technically difficult than standard lobectomy; therefore, proficient knowledge of anatomical variations becomes increasingly important for the general thoracic surgeon. Here, we report the case of a patient with a bronchial variation in the posterior segment (S^2^) of the right upper lobe who underwent thoracoscopic segmentectomy and lymph node sampling for lung cancer and a literature review.

## Case presentation

**Fig. 1:**
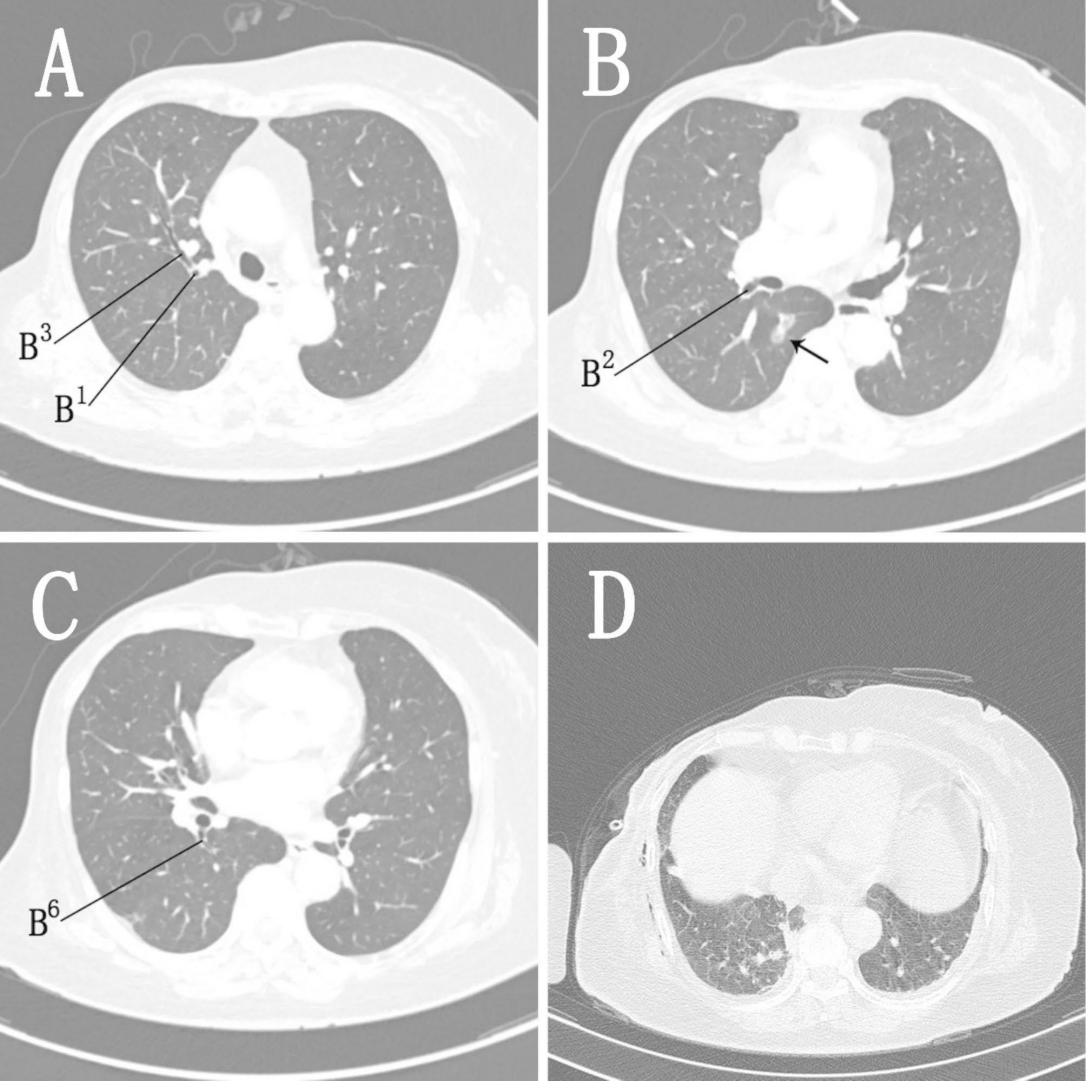
A + B + C, Preoperative lung CT; a mixed ground-glass nodule (arrow) measuring approximately 17 mm × 8 mm with a CT value of -700 HU was observed in the posterior segment of the right upper lobe. D, Postoperative lung CT. CT, computed tomography

A 74-year-old female patient presented with an abnormal shadow on chest computed tomography (CT) at a medical checkup and subsequently visited our hospital. Chest CT showed a 17 mm × 8 mm ground-glass opacity with approximately 30% solid component in the right superior segment (S^6^) (Fig. 1A-C). The patient had undergone radical treatment of right breast cancer staged at pT2N0M0 IIA, followed by four cycles of postoperative adjuvant chemotherapy 11 years prior. All other medical history was unremarkable.

**Fig. 2:**
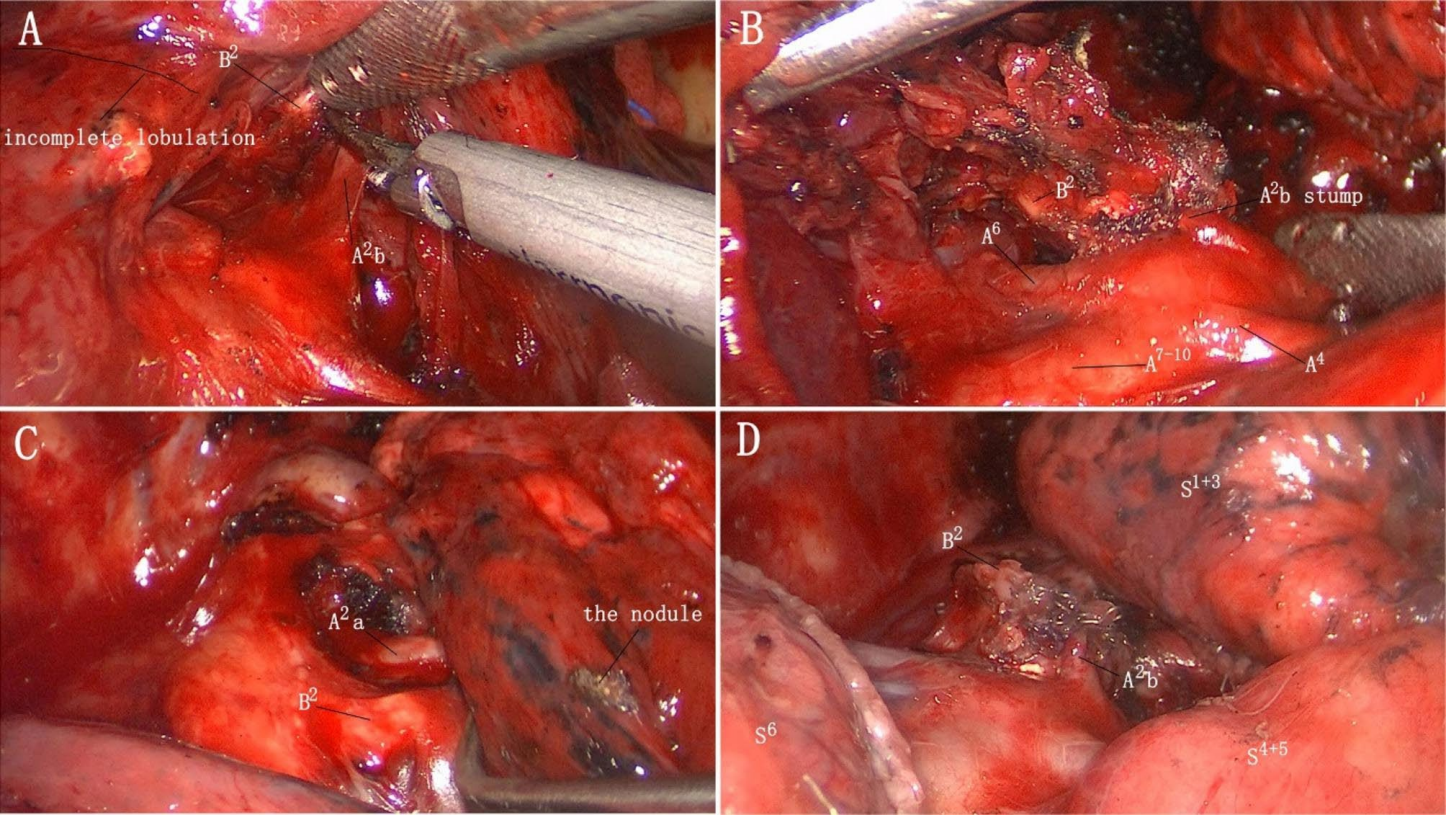
Patient anatomy during surgery

Relevant examinations were performed after admission, and no obvious contraindications to surgery were observed. Because it was difficult to palpable the lesion and the location of lesion was closed to the bronchus, a thoracoscopic right S^6^ segmentectomy was planned. We performed thoracoscopic surgery using two ports. We made a 2-cm incision in the 7th intercostal space of the right midaxillary line and a 4-cm incision in the 4th intercostal space of the right anterior axillary line as the observation and operating holes, respectively. Pleural adhesions were observed throughout the thoracic cavity perioperatively. The right lung was found to be divided into three lobes after being released. The horizontal and posterior oblique fissures were poorly developed, and the intersegmental plane could not be distinguished. Digital palpation indicated that the nodule was located in a high position, and lifting the right upper lobe revealed the suspected location of the nodule in the upper lobe. The interlobar fissures were separated, and the posterior segmental and superior segmental arteries were located following the pulmonary trunk arteries (Fig. 2A,B). After labelling the nodule on the pleural surface, multiple comparisons were performed, and the nodule was eventually found in the S^2^ of the right upper lobe (Fig. 2C). So the recurrent and ascending arteries were dissociated and resected. Then, the posterior segmental bronchus (B^2^) was exposed and transected. Finally, the right lung was reventilated with pure oxygen and the intersegmental plane was clear after 20 min. The intersegmental plane was divided along the inflation-deflation line using the endostaplers; thus, resection of the S^2^ of the right upper lobe was completed (Fig. 2D). The tumor was 3 cm away from the incisal margin. As intraoperative pathology analysis revealed minimally invasive adenocarcinoma (MIA), hilar lymph node sampling was performed. On postoperative day 2, the right lung was completely redilated on CT (Fig. 1D), and postoperative pathological examination revealed MIA with negative surrounding lymph nodes.

**Fig. 3:**
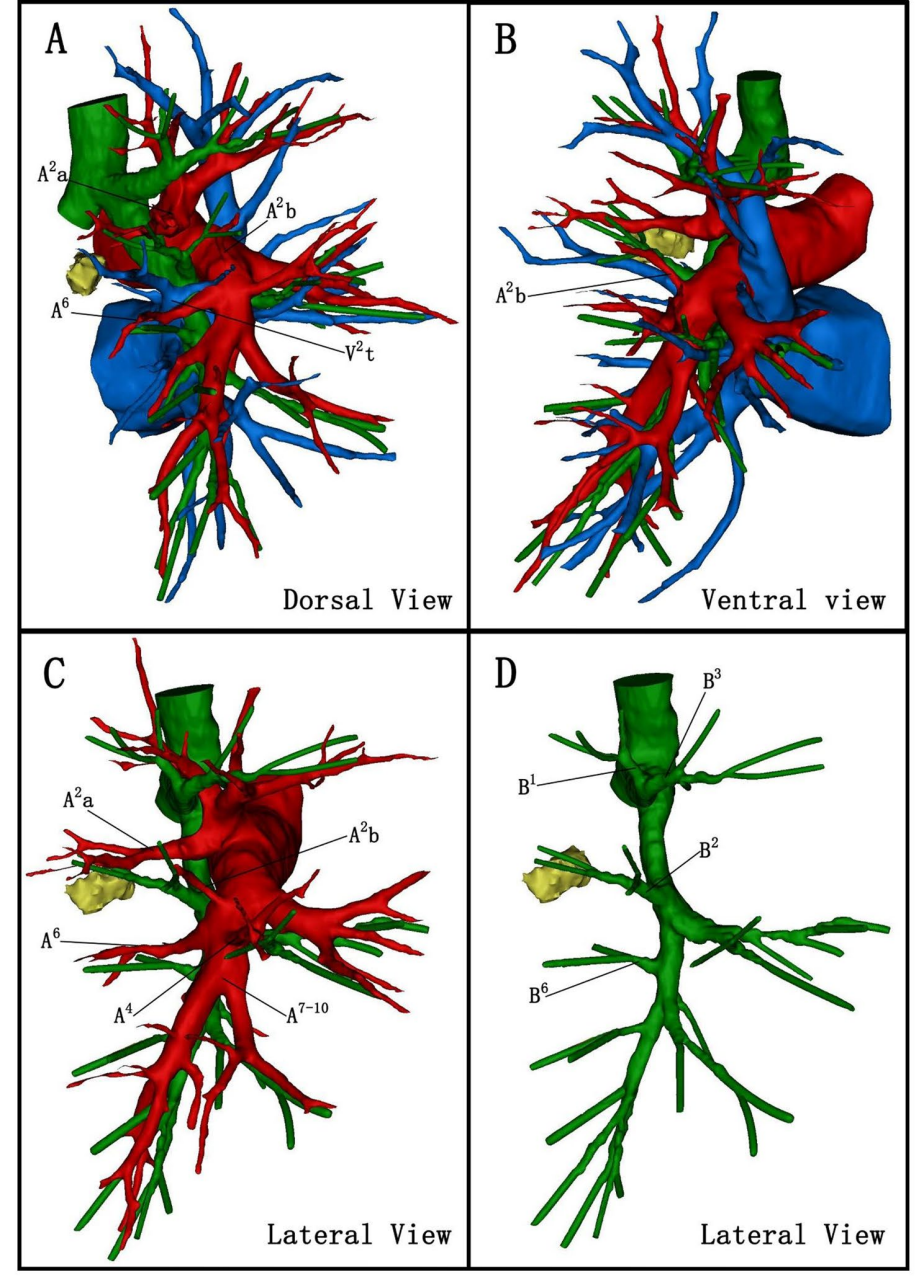
Three-dimensional reconstruction of the patient. The apical segmental bronchus (B^1^) and anterior segmental bronchus (B^3^) originate from the right upper lobe bronchus, while the posterior segmental bronchus (B^2^) originates from the bronchus intermedius. A^2a^ originates from the posterior recurrent branching artery, A^2b^ originates from the posterior ascending branching artery, and there is no central vein in the right upper pulmonary vein and only a V^2t^, which enters the left atrium from the posterior aspect of the right lower lobe bronchus. A, dorsal view; B, ventral view; C + D: lateral view

We reviewed lung CT images and performed three-dimensional (3D) reconstructions using Mimics Medical 21.0 software postoperatively. It revealed that the B^2^ originated from the bronchus intermedius, the posterior segmental artery (A^2^) of the right upper lung lobe bifurcated into the A^2a^ and A^2b^ branching from the recurrent and ascending arteries, respectively, and the right superior pulmonary vein had no central vein but a posterior intrasegmental vein ( V^2t^ ) that travelled below the S^2^ (Fig. 3).

## Discussion and conclusions

Pulmonary segmentectomy is one of the most discussed topics in thoracic surgery since it requires an accurate understanding of the targeted lung segment to be successful [2]. However, lung segments frequently exhibit anatomical variations, particularly in the bronchi of the right upper lobe [3, 4]. Here, we reported a case of bronchial variation in the right upper lobe and reviewed the literature on this variation to provide clinical guidance for pulmonary segmentectomy.

Trifurcated and bifurcated bronchi are common anatomical variations in the right upper lobe. The bifurcated type can be classified into the simple (B^1 + 2^, B^3^; B^1 + 3^, B^2^; and B^2 + 3^, B^1^) or intersecting subtypes (B^1a + 2^, B^1b + 3^ and B^1 + 2a^, B^2b + 3^) whereas the quadrifurcated type is open in the right upper lobe and relatively rare. Contrary to the numerous variations of lobar or segmental bronchial subdivisions, abnormal bronchi originating from the trachea or main bronchi are rarer. For example, the tracheal bronchus, which is a distal or proximal displacement of the bronchus in the right upper lobe, is a variant of proximal bronchial displacement that arises directly from the trachea or primary bronchi, proceeding towards the right upper lobe. Conversely, distal displacement of the bronchus in the right upper lobe has been reported [5]. In very rare cases, a segmental bronchus of the lung may be ectopic to the distal or proximal bronchi [6, 7]. Yaginuma previously reported 15 cases of apical segmental bronchus (B^1^), which is ectopic to the lateral wall of the trachea or the right main bronchus (Right B^1^ Type), and 7 and 11 cases of B^2^ and anterior segmental bronchus (B^3^), respectively, originating directly posterior to the bronchus intermedius (Right B^2^ Type and Right B^3^ Type) [7]. In the Right B^2^ and Right B^3^ Types, abnormal lung lobulations and top pulmonary veins were frequently noted. For the Right B^2^ Type, all patients had an incomplete lobulation between the upper and lower lobes; however, no abnormality of the pulmonary artery was observed. We reviewed these anatomical types of bronchi in the right upper lobe (Table 1) and described a Right B^2^ Type that was misdiagnosed.

**Table 1:** Previously reported anatomical variations of the bronchi in the right upper lobe

	Right upper lobe	Inspection method	Author, Year
Normal findings and most common bronchial variations	Trifurcation B^1^, B^2^, B^3^	3DCT; Bronchography	Nagashima et al. (2015) [3]; Gonlugur et al. (2005) [8]
	Bifurcation B^1 + 2^, B^3^	3DCT; Bronchography	Nagashima et al. (2015) [3]; Gonlugur et al. (2005) [8]
	Bifurcation B^1 + 3^, B^2^	3DCT; Bronchography	Nagashima et al. (2015) [3]; Gonlugur et al. (2005) [8]
	Bifurcation B^2 + 3^, B^1^	3DCT; Bronchography	Nagashima et al. (2015) [3]; Gonlugur et al. (2005) [8]
	Bifurcation B^1a + 2^, B^1b + 3^	3DCT; Bronchography	Nagashima et al. (2015) [3]; Zhang et al. (2021) [9]
	Bifurcation B^1 + 2a^, B^2b + 3^	3DCT; Bronchography	Nagashima et al. (2015) [3]; Zhang et al. (2022) [10]
	Quadrifurcation	3DCT; Bronchography	Nagashima et al. (2015) [3]; Gonlugur et al. (2005) [8]
Rare variations	Tracheal bronchus	Thoracoscopy; Bronchography	Yurugi et al. (2012) [11]; Martín-Ruiz et al. (2021) [4]
	Right upper lobe bronchus ectopic to the intermediate bronchus	3DCT	Huang et al. (2020) [5]
	B^1^ ectopic to the lateral wall of the trachea or the right main bronchus (Right B^1^ Type))	CT	Yaginuma (2020) [7]
	B^2^ ectopic to the intermediate bronchus (Right B^2^ Type)	CT	Yaginuma (2020) [7]
	B^3^ ectopic to the intermediate bronchus (Right B^3^ Type)	3DCT	Ghaye et al. (2001) [6]; Yaginuma (2020) [7]

B^1^ = apical segmental bronchus; B^2^ = posterior segmental bronchus; B^3^ = anterior segmental bronchus; 3DCT = three-dimensional computed tomography; CT = computed tomography

The patient we reported also had an incomplete lobulation between the right upper and lower lobe, and no abnormality of the pulmonary artery was found. The right superior pulmonary vein had no central vein but a V^2t^ that travelled below the S^2^. In this case, the patient had a nodule located in the S^2^ rather than the S^6^. The B^2^ originated from the bronchus intermedius, and no significant abnormalities were found in the apical and anterior segmental bronchi, whereas the bronchus, which was originally believed to be a subsuperior segment (S*), was the superior segmental bronchus (B^6^). Therefore, a right posterior segmentectomy was performed.

Although the overall surgical procedure was uneventful, it was time-consuming and difficult. However, if this anatomical variation had been better understood before surgery, the surgical approach selection and the intersegmental plane identification would have been more streamlined, and the duration of surgery could have been shortened.

Despite the rarity of bronchial displacement incidence, it may present the risks of bronchovascular injury and difficulties in different lung ventilation processes. Therefore, identifying the displaced bronchi and associated pulmonary lobulations and vascular course abnormalities is important before anatomic segmentectomy. Furthermore, 3D imaging technology can help reveal anatomical variations promptly, particularly the rare types of bronchial branching, and is effective at improving the accuracy and safety of pulmonary segmentectomy.

## Data Availability

The datasets used and/or analysed during the current study are available from the corresponding author on reasonable request.

## Abbreviations

A^2^: posterior segmental artery
A^4^: lateral segmental artery
A^6^: superior segmental artery
A^7–10^: basal segmental artery
V^2t^: posterior intrasegmental vein
B^1^: apical segmental bronchus
B^2^: posterior segmental bronchus
B^3^: anterior segmental bronchus
B*: subsuperior bronchus
B^6^: superior segmental bronchus
S^2^: posterior segment
S^6^: superior segment
MIA: minimally invasive adenocarcinoma
3D: three-dimensional
CT: computed tomography
